# Promotion of Meaning in Life and Wellbeing Among University Students During the COVID-19 Pandemic *via* a Service-Learning Subject

**DOI:** 10.3389/fpubh.2022.924711

**Published:** 2022-06-21

**Authors:** Xiaoqin Zhu, Wenyu Chai, Daniel T. L. Shek, Li Lin

**Affiliations:** ^1^Department of Applied Social Sciences, The Hong Kong Polytechnic University, Hong Kong, China; ^2^Department of Applied Psychology, Lingnan University, Hong Kong, China

**Keywords:** meaning in life, life satisfaction, service-learning, university students, learning effectiveness

## Abstract

Utilizing the principle of “learning by doing,” service-learning (SL) course provides a platform for university students to apply academic knowledge in serving the community, reflecting on the serving experiences, deepening their understanding of the knowledge, and further improving their competence, responsibility, wellbeing, and meaning in life (MIL). This study reported university students' changes in psychological wellbeing (positive youth development attributes), subjective wellbeing (life satisfaction), and MIL after taking a SL subject during the COVID-19 pandemic through a one-group pretest-posttest design. Based on the data collected from 229 students (mean age = 20.86 ± 1.56 years, 48.0% females), repeated-measures multivariate general linear model (GLM) analyses revealed that students showed significant positive changes in wellbeing and MIL. In addition, pretest MIL scores positively predicted posttest scores of the two wellbeing measures but not vice versa. As predicted, improvement in MIL among students was closely associated with the positive changes in both psychological and subjective wellbeing measures. These findings suggest that SL participation during the pandemic may promote students' life meaning and foster their wellbeing. Furthermore, MIL and wellbeing may improve simultaneously, and MIL enhancement may further contribute to improvement in psychological and subjective wellbeing. The findings further prove that SL is an effective pedagogy in higher education settings in promoting youth positive development.

## Introduction

### Promoting Students' Meaning in Life and Wellbeing in Higher Education

In recent years, promoting university students' positive and holistic development has become an increasing concern in higher education. University students are in emerging adulthood, which can be considered a transition from late adolescence to adulthood. When entering university, students need to adapt to a less structured learning environment, fulfill demanding academic requirements, and become more independent in more complicated social and learning environments. They also face the developmental challenge of taking on new civic roles and greater responsibilities and finding meaning and purpose in these roles (1). Those who ineffectively cope with stress and challenges may suffer from dysfunctional outcomes, such as loss of meaning, emotional distress, low wellbeing, and behavioral problems (2–4). This concern for university students' development has been further intensified by the spreading of COVID-19 since early 2020, which has a significant impact on higher education. Due to the interruption of normal university life, the sudden transition to online studies, and the social distancing measures, university students tend to feel more depressed, stressed, and less satisfied (5, 76). Students may also feel a loss of meaning due to social isolation as interpersonal relationships and connections with others are important factors contributing to their sense of meaning (6, 7).

Despite the challenges and difficulties, youths have the potential to develop and flourish. This possibility has been increasingly emphasized by researchers and educators with the paradigm shift from focusing on treating mental illness and psychological problems to cultivating youths' strengths, potentials, and wellbeing (8). In line with this paradigm shift, there has also been a trend highlighting the promotion of positive functioning and holistic development among university students (80). Among different indicators, meaning in life (MIL) and wellbeing are two essential indicators being increasingly highlighted. Thus, how to promote MIL and wellbeing among university students becomes vital in current higher education settings.

MIL refers to “people's beliefs that their lives are significant and that they transcend the ephemeral present” (9). It includes “a sense of coherence” and “a sense of purpose,” providing people with motivation and directions and helping them realize their worth and values, thus eventually leading to better human functioning (9). Research findings showed that higher MIL is closely associated with less psychological distress, higher subjective wellbeing (e.g., life satisfaction), and better psychological functioning such as emotional competence and hope (10–13). Conversely, a lack of MIL is linked to a broad range of psychological disorders (e.g., anxiety, depression, stress, addiction) and even disruptive behaviors such as substance abuse and suicidal behavior (11, 14, 15).

A concept related to MIL is purpose in life, which has been considered “a stable and generalized intention to accomplish something that is meaningful to the self and leads to engagement with some aspect of the world beyond the self” (16). As the two constructs are conceptually relevant, some scholars even used the terms “purpose in life” and MIL interchangeably (17–19). Nevertheless, some researchers proposed that they are different. While MIL refers to the significance of one's life such that life matters and is worthwhile, purpose of life may vary among individuals, from a focus on benefiting self or emphasis on others' needs to the discovery of the world (20). As Heng et al. (21) remarked, “the meaning of life is to find one's gift. The purpose of life is to use this gift for the benefit of both self and others” (pp. 303–304). According to this conceptualization, purpose in life is an important contributor to MIL, which is consistent with Steger's conceptualization of MIL. In this study, we used the term MIL according to Steger's definition.

Wellbeing, which refers to an individual's “optimal psychological functioning and experience” (22), has also become an increasing concern of researchers, university administrators, and healthcare professionals as students need to excel in academic domains and beyond (23). Wellbeing is conceived as including two aspects: psychological and subjective wellbeing. Psychological wellbeing adopts the perspective of eudaimonia and emphasizes a set of psychological wealth (e.g., personal growth, competence, social connections) that underlies human fully functioning (24, 25). Subjective wellbeing, on the other hand, concentrates on hedonic experiences in the form of pleasant feelings in the physical, emotional, spiritual, and cognitive domains (22). These two aspects have been operationally indexed by different measures. For example, positive youth development (PYD) competence (e.g., emotional and social skills and resilience) are considered measures of psychological wellbeing (26, 27) while life satisfaction has been frequently used to indicate subjective wellbeing (22, 28). Noteworthy, in some models, such as those proposed by Ryff and Waterman and their collaborators (29, 30), MIL is one of the components of psychological wellbeing. To avoid conceptual overlap, we focused on PYD competence as an indicator of psychological wellbeing that reflects an individual's potential and growth (25, 79). Empirically speaking, higher levels of psychological and subjective wellbeing are associated with better academic performance, and fewer emotional and behavioral problems (4, 31).

Recognizing the importance of MIL and wellbeing among university students, higher education institutions worldwide have devoted considerable efforts and resources to promoting students' MIL and wellbeing through designing and implementing credit-bearing curriculums and/or non-credit-bearing youth programs or services. For example, a wide range of higher-impact educational practices, such as collaborative projects, learning communities, community-based learning, and service-learning, have been effectively incorporated into universities' general education curriculums to facilitate students' growth in psychological and spiritual domains in addition to academic achievement [e.g., (32–34)]. Among different pedagogical approaches, service-learning (SL) is a form of “higher-impact educational practice” that aims to facilitate students' overall healthy development (33). It is believed that serving others and contributing to community welfare through SL help students gain a sense of meaning and foster wellbeing (35).

### Promoting Students' MIL and Wellbeing Through SL Pedagogy

Grounded in experiential learning theory (36, 37), SL is a “learning by doing” pedagogy. In doing SL, students not only learn academic theories and concepts but also apply their knowledge and skills in providing community services and then further reflect on the serving experiences (38, 39). In essence, students go through a learning circle of doing, reflecting, learning, and improving in SL (36, 40). This learning circle enables them to deepen their understanding of the academic concepts, put theories into practice, make contributions to the community, and enhance a wide range of generic competence (e.g., problem-solving, caring, and responsibility). Unlike volunteering activities that typically aim to benefit service recipients and do not have well-articulated educational components for service providers, SL holds a core philosophy of “reciprocity,” or in other words, providing benefits for both service providers and service recipients (38).

By committing to satisfying community needs and fulfilling their own educational goals, students have opportunities to reflect on the purpose of learning and serving during SL. Zhang et al. (41) remarked that “to accomplish one's aspirations and actualize one's potential” and “to contribute to society and to adhere to moral principles” are two important sources of MIL among university students. Obviously, SL participation provides such foundations for forming MIL. As argued by Welch and Koth (42), students may experience a sense of connection with self, others, and the whole community in SL that contributes to their reflection on and construction of MIL. According to Moran (43), “life purpose” is a “correlational outcome” and “mediator” of SL participation as it helps students to reflect on their professional and civil roles in community settings. Empirical evidence is in line with these theoretical propositions. For example, Barrett (35) found that SL participation promoted the spiritual development of college students. Another study showed that SL experience leads to changes in life meaning among student-teachers (44). Moreover, a recent study demonstrated that a sense of belonging and supportive relationships derived from the SL process predicted the development of MIL in students (45).

The unique learning process involved in SL is also expected to unleash students' potential, and enhance their overall development, thus improving wellbeing (39). Empirical studies have demonstrated positive impacts of SL on university students' psychological wellbeing (e.g., self-efficacy, leadership, PYD qualities) and subjective wellbeing (e.g., life satisfaction) (46, 47). However, there is also research showing a limited increase or even a decrease in psychological wellbeing or subjective wellbeing after joining a SL program [e.g., (48, 49)]. Possible explanations include that students might have a more “realistic and objective view of themselves” after the SL experience (49), or they may feel unhappy after understanding the predicament and problems of underprivileged people in the SL process (48). Nevertheless, meta-analyses generally suggest that students would achieve significant gains in multiple areas (e.g., self-worth, social skills, academic learning, and civic engagement) after joining SL programs (40, 50). Despite the general positive evaluation findings in the existing literature, more research is needed to clarify the equivocal findings and further understand the impacts of SL on students' wellbeing during the pandemic.

### Research Gaps

Existing studies on SL effectiveness are based primarily on the “traditional” face-to-face SL. Hence, there is a need to understand how SL participation is related to MIL and wellbeing during the COVID-19 pandemic when there is a significant change in SL implementation mode. With the advancement of information technology in recent decades, SL programs in some universities have been implemented through a virtual mode during the pandemic (51–53). However, traditional SL involves much interpersonal interaction and experiential learning, which might not be effectively achieved through virtual SL. In essence, it is hard for students to provide direct on-site face-to-face services to people in need when everyone has to follow mandatory social distancing measures. For example, students were found to feel highly stressed in conducting online SL activities due to unfamiliarity, a feeling of helplessness, and being out of control during the pandemic; students also had low motivation in an online environment as they did not know how the services they provided may influence the service recipients (54). While online teaching and remote non-face-to-face services are feasible during the COVID-19 pandemic, the benefits of participating in SL in such a way become questionable.

Nevertheless, a handful of available studies showed that online SL is still beneficial. One study revealed that students taking online SL programs during the pandemic showed similar gains in leadership attributes, PYD qualities (e.g., self-identify and social competence), and life satisfaction as did those students taking traditional face-to-face SL programs pre-pandemic (52). Leung et al. (55) also reported that students demonstrated significant improvements in their competence after completing an online SL program during the pandemic. In a case study on virtual SL activities during the pandemic, students expressed that they gained spiritual development such as a sense of belonging, gratitude, and love (53). As evaluation studies on online SL are not rich and the findings are not entirely consistent, whether and how online SL may influence students' MIL and wellbeing deserve further investigation.

Moreover, while previous studies have assessed MIL or wellbeing as outcomes of SL participation, limited attention has been devoted to the relationship between MIL change and wellbeing change. SL may impact students' MIL and wellbeing simultaneously, as students may achieve satisfaction, a sense of growing, and life meaning at the same time when they engage in SL. It is also possible that MIL and wellbeing reinforce each other. MIL and wellbeing have been found to be robustly associated with each other (10). On the one hand, meaning plays a central role in an individual's life that makes one “being directed and motivated by valued goals” (56). Thus, MIL may serve as a prerequisite for living a good and happy life (i.e., psychological and subjective wellbeing). Indeed, MIL has been long considered one of the key components of psychological wellbeing (29, 57). On the other hand, feeling good (subjective wellbeing) may broaden one's cognitive and emotional scope and capabilities (58), which are essential resources for an individual to pursue meaningful goals. As aforementioned, some elements of psychological wellbeing, such as self-development and self-actualization are important building blocks of MIL (41). Despite all these possibilities, to our best knowledge, no studies to date have empirically tested these mechanisms.

### The Present Study

To address the above-mentioned research gaps, the present study endeavored to investigate whether students had positive changes in their wellbeing and MIL after taking a SL subject entitled “Promotion of Children and Adolescent Development” (“Promotion Subject”) offered by the authors' university during the pandemic. In this study, wellbeing measures included both psychological and subjective wellbeing. Based on previous findings suggesting beneficial impacts of SL (55, 59), we hypothesized significant positive changes in students' wellbeing (Hypothesis 1) and MIL (Hypothesis 2) after their SL participation. Given the possibility that MIL and wellbeing may reinforce each other and change simultaneously, we expected reciprocal associations between MIL and wellbeing (Hypothesis 3). We also expected a parallel improvement in wellbeing and MIL (Hypothesis 4), which can be indicated by a significant inter-correlation between students' changes in wellbeing and MIL (27).

## Methods

### Overview of the “Promotion Subject” During the COVID-19 Pandemic

In the academic years of 2019–2020 and 2020–2021, the 3-credit “Promotion Subject” was offered to students in two consecutive semesters (i.e., the first and second semesters) at the authors' university. This SL course was designed based on both developmental theories and SL approaches, such as the PYD and ecological models as well as the experiential learning framework mentioned earlier. After taking this course, students would be able to (i.e., the intended learning outcomes):

a) “understand different perspectives of child and adolescent development and apply the concepts in understanding the needs and potentials of underprivileged children and adolescents in the community,”
b) “integrate knowledge on child and adolescent development into real-life situations through critical thinking and service delivery,”
c) “apply the knowledge and skills they have acquired in university education to deal with complex issues in the service setting and pursue continual self-improvement,”
d) “reflect on their roles and responsibilities both as a professional in their chosen discipline and as a responsible global citizen,”
e) “develop the sense of care and compassion toward other people, especially the underprivileged children and adolescents in the community,” and
f) “enhance psychosocial competence such as self-confidence, innovative problem solving, decision-making capabilities, interpersonal skills, self-leadership, and teamwork.”

To achieve these outcomes, students were required to spend 135-h study effort in this course. First, students needed to complete a 10-h e-learning module that introduced the basic concepts and benefits of SL and promoted students' positive attitudes toward service projects. Second, there were 30 h of lecturing (10 lessons, 3 h per lesson) on the theoretical foundations of the subject (e.g., strength-based approaches, PYD models, SL theory), characteristics of service targets (i.e., underprivileged children and adolescents from single-parent or poor families or with adjustment problems), principles, attitudes, and skills in serving. Third, students provided 40-h direct services. Finally, students spent 37 h on service preparation and post-service integration and reflection, and 18 h on reading and self-study. During the course period, students completed the e-learning module, attended the seven lessons, and worked out service plans in groups (5–6 students per group) in the first semester.

Each group of students carried out their services across two semesters, mainly from October to March next year. More specifically, students provided services to underprivileged children and adolescents (e.g., those from single-parent or poor families or with adjustment problems) with the purpose to promote their academic and holistic development. The services mainly took the form of workshops on both academic and non-academic knowledge and skills, as well as large-scale activities such as university visits and day camps. Three lessons were scheduled in the second semester to facilitate students' reflection on and consolidation of their service experience, with the first two during the service provision period and the last one after the completion of the service.

Given the campus lockdown and related renovation from November to December 2019 and the COVID-19 pandemic since early 2020, online teaching and learning mode was adopted most of the time when the “Promotion Subject” was offered in the 2019–2020 and 2020–2021 years. Since early February 2020 (i.e., the outbreak of COVID-19), the mode of service has also been adjusted and changed to non-face-to-face service provided through different online platforms and tools (e.g., online workshops, video teaching, and virtual university campus tour). In other words, some of the services in the 2019–2020 year and all the services in the 2020–2021 year were non-face-to-face.

### Study Design

To achieve our reach aims and test the hypotheses, this study adopted a pre-experimental research design, i.e., a one-group pretest-posttest design. Although this research design suffers intrinsic limitations due to the lack of a control group (60), it is practically preferred. Sophisticated experimental research with control groups and random grouping is very demanding in financial and human resources, thus it is practically very difficult to be employed in educational contexts. As remarked by Thyer (61), “you must control for the most relevant threats to internal validity” or “you must randomly assign clients to various control and experimental groups” are myths in assessing educational impacts. As such, scholars still regard the one-group pretest-posttest design as a valuable research design that can be meaningfully applied in educational and other human service settings, such as the social work field (61, 62). This research design has been widely adopted in studies examining educational effectiveness [e.g., (27, 55, 63)].

### Participants and Procedures

The pretest-posttest assessment was one of the evaluation components incorporated in the “Promotion Subject,” and the evaluation plan had been approved by the Institutional Review Board (and its Delegate) at the authors' university. Students enrolled in the subject were invited to complete the same survey at the beginning of the first lecture (the pretest) and after completing all services (the posttest). A total of 92 (43.5% female students, mean age = 20.84 ± 1.37 years) and 137 (50.7% female students, mean age = 20.87 ± 1.68 years) students were matched for the two academic years, respectively. The mean age of the whole matched sample (*N* = 229) was 20.86 ± 1.56 years and 48.0% of the students were female.

### Instruments

The pre-test and post-test questionnaires contained three major measures, including MIL, psychological wellbeing indexed by multiple PYD competence reflecting healthy functioning in different domains, and subjective wellbeing indexed by life satisfaction reflecting an overall subjective assessment of one's quality of life.

PYD competence was assessed by ten subscales of the “Chinese Positive Youth Development Scale” (64), with each subscale assessing one PYD attribute, such as “emotional competence,” “resilience,” “social competence,” “clear and positive identity,” and so forth. These attributes were grouped into three higher-order factors, including (a) “cognitive and behavioral competence” covering three subscales (“cognitive competence,” “behavioral competence,” and “self-determination”); (b) “positive identity” covering two subscales of “clear and positive identity” and “belief in future”; and (c) “general PYD competence” covering five subscales (e.g., “social competence,” “emotional competence,” and “resilience”). Students rated their PYD competence on a 6-point reporting scale (“1” = “strongly disagree,” “6” = “strongly agree”). In the current study, we used four composite scores computed based on the three higher-order factors and a total score of all the ten subscales (i.e., the total average score). These scales demonstrated adequate internal consistency in the current study (see Table 1).

**Table 1:** Reliability and overall changes in different outcome indicators between the pretest and posttest.

**Variables**	**Pretest**		**Posttest**		***F* value**	***η^2^p***
	***M* (*SD*)**	**α (Mean inter-item correlation)**	***M* (*SD*)**	**α (Mean inter-item correlation)**		
Positive Youth Development					11.56^***^^,^^a^	0.20
Cognitive and behavioral competence	4.62 (0.66)	0.91 (0.54)	4.93 (0.61)	0.92 (0.56)	46.05^***^	0.19
Positive identity	4.42 (0.82)	0.86 (0.54)	4.67 (0.84)	0.89 (0.63)	24.89^***^	0.11
General positive youth development competence	4.55 (0.57)	0.88 (0.35)	4.75 (0.58)	0.89 (0.40)	27.04^***^	0.12
Total score of positive youth development competence	4.54 (0.60)	0.95 (0.40)	4.79 (0.60)	0.96 (0.47)	40.63^***^	0.17
Life satisfaction	3.95 (0.98)	0.90 (0.66)	4.21 (1.06)	0.94 (0.75)	15.26^***^	0.07
Meaning in life	4.00 (0.93)	0.89 (0.64)	4.14 (0.88)	0.85 (0.56)	4.12^*^	0.02

a *Adjusted bonferroni value = 0.013*.

* *p < 0.05*.

*** *p < 0.001*.

Life satisfaction was assessed by the Chinese version of the “Satisfaction with Life Scale” (65), which included five items, such as “the conditions of my life are excellent” and “In most ways, my life is close to my ideal.” Students reported their agreement with these statements also on a 6-point scale (“1” = “strongly disagree,” “6” = “strongly agree”). The scale showed adequate internal consistency in this study (see Table 1).

MIL was measured by the 5-item subscale of “presence of meaning” in the “Meaning in Life Questionnaire” (12), which has been translated into Chinese and widely employed in different Chinese samples (2, 28). Students reported the degree of their agreement with the five statements about the presence of meaning (e.g., “My life has a clear sense of purpose”) on a 6-point scale (“1” = “strongly disagree,” “6” = “strongly agree”). In the current study, the scale demonstrated adequate internal consistency (see Table 1).

### Data Analysis

Before formal data analysis, the internal consistency of each measure was checked. To ensure correct longitudinal comparisons, the longitudinal invariance tests (including configural, metric, and scalar invariance) were performed for each measure following the procedures in previous studies (66, 81). In the present study, the structures of PYD competence (i.e., a latent PYD factor indicated by the three higher-order factors), life satisfaction (i.e., latent life satisfaction indicated by the five items), and MIL (i.e., latent MIL indicated by the five items) were longitudinally invariant. Specifically, the changes in “comparative fit index” (i.e., ΔCFI) were <0.01 in both metric (PYD: ΔCFI = 0.002; life satisfaction: ΔCFI = 0.006; MIL: ΔCFI = 0.004) and scalar invariance tests (PYD: ΔCFI = 0.005; life satisfaction: ΔCFI = 0.001; MIL: ΔCFI = 0.004). The changes in “root mean square error of approximation” (i.e., ΔRMSEA) were <0.015 in both metric (PYD: ΔRMSEA = 0.001; life satisfaction: ΔRMSEA = 0.009; MIL: ΔRMSEA = 0.006) and scalar invariance tests (PYD: ΔRMSEA = 0.012; life satisfaction: ΔRMSEA = 0.003; MIL: ΔRMSEA = 0.005).

A series of repeated-measures multivariate general linear model (GLM) analyses were performed using SPSS Version 25.0 (IBM Corp., Somers, NY, USA) to investigate whether there were significant changes in students' PYD competence, life satisfaction, and MIL after taking the “Promotion Subject” (i.e., from the pretest to posttest). For PYD competence, the aforementioned four composite scores were used as outcome indicators and the Bonferroni procedure was employed to reduce inflated Type I error. If the omnibus time effect (pretest vs. posttest) for PYD competence was significant, follow-up univariate analyses would be performed for each PYD composite score. To check whether there were any differences regarding students' changes across the academic year and gender, we first run the repeated-measures GML analyses with the academic year and gender as two between-subject variables. As there were no significant main effects of the two variables nor the interactions (*F* ranged between 0.003 and 2.29, *p*s > 0.05). The final analyses used the whole sample (*N* = 229) while treating academic year, gender, and age as control variables.

**Figure 1:**
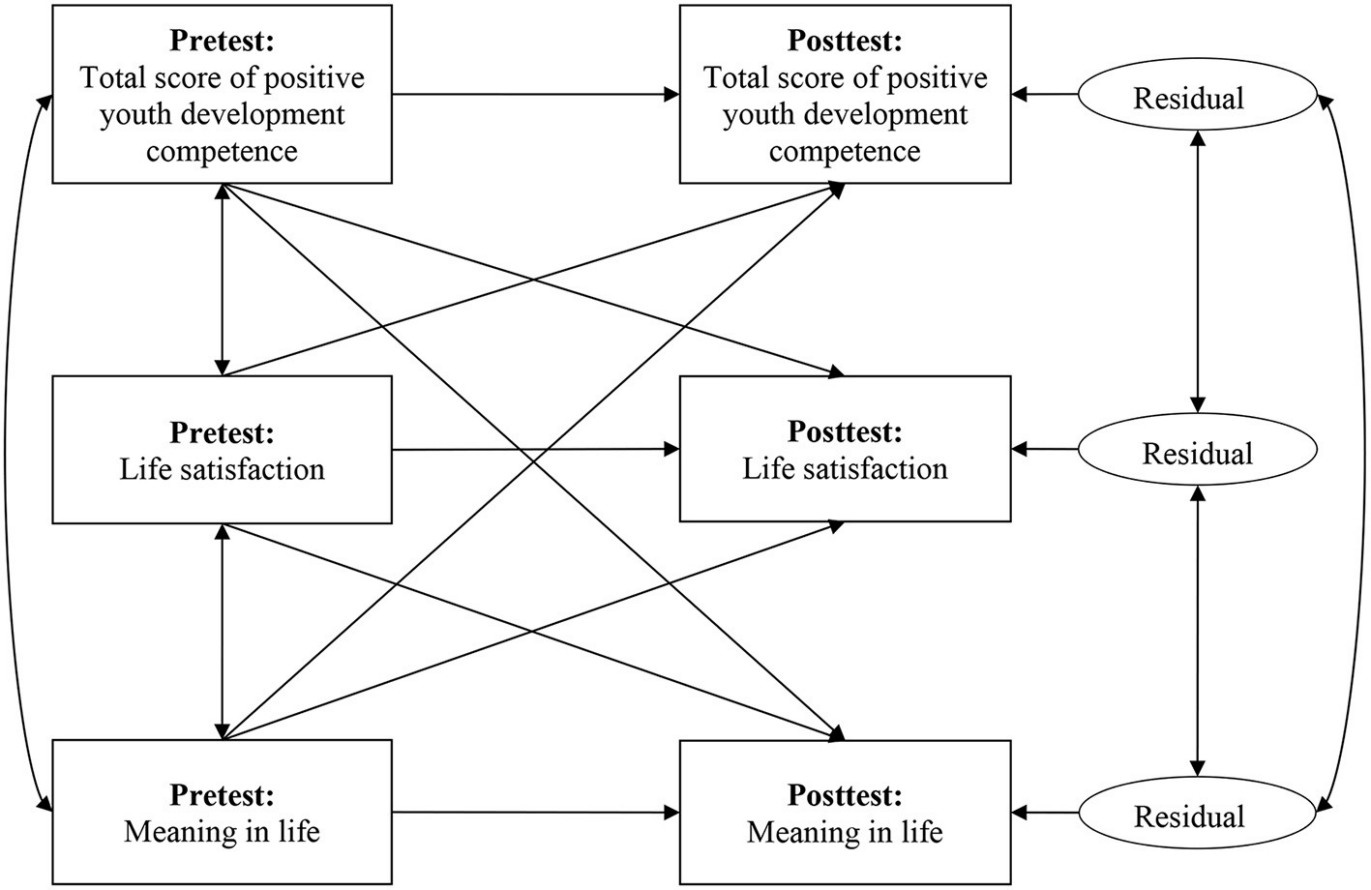
Cross-lagged panel model among positive youth development, life satisfaction, and meaning in life in pretest and posttest.

A cross-lagged path analysis was also performed using AMOS Version 25.0 (IBM Corp., Somers, NY, USA) to investigate the cross-lagged effects among PYD competence (the total PYD score was used), life satisfaction, and MIL as well as the relationship between changes in these outcome measures. As presented in Figure 1, there were four types of paths in the cross-lagged path model, including (1) three concurrent associations among the pretest scores; (2) three autoregression paths modeling the temporal stability; (3) six cross-lagged effects showing the effect of one variable's pretest score on a different variable's posttest score; and (4) the correlated change indicated by the association between every two variables' posttest scores. As the first three types of associations between the variables were statistically controlled in the model, a correlated change can be interpreted as a reliable estimation of how individual change in one variable over time was associated with the change in another variable (27, 67, 68). More specifically, the three correlated changes modeled among the three outcome measures answered the question as to whether students' wellbeing and MIL developed simultaneously (e.g., students' life satisfaction increased as their MIL increased). Because all variables in the cross-lagged model were associated with each other, the degree of freedom was zero and the model was a saturated one with perfect fitness.

## Results

As shown in Table 1, there were significant positive changes from the pretest to the posttest in PYD competence (omnibus effect: *F* = 11.56, *p* < 0.001, $\eta_{p}^{2}$ = 0.20), life satisfaction (*F* = 15.26, *p* < 0.001, $\eta_{p}^{2}$ = 0.07), and MIL (*F* = 4.12, *p* < 0.05, $\eta_{p}^{2}$ = 0.02). For the PYD competence, follow-up tests revealed that students gained positive changes in all four compositive measures (*F* ranged between 24.89 and 46.05, *p*s < 0.001, $\eta_{p}^{2}$ ranged between 0.11 and 0.19). In general, students demonstrated significant improvements in their psychological wellbeing, subjective wellbeing, and MIL, thus supporting Hypotheses 1 and 2.

Regarding the cross-lagged effects (see Table 2), MIL at pretest significantly and positively predicted posttest PYD competence (β = 0.17, *p* < 0.05) and life satisfaction (β = 0.19, *p* < 0.05), while other cross-lagged effects were not significant. Thus, Hypothesis 3 (i.e., reciprocal relationships between wellbeing and MIL) was not supported.

**Table 2:** Results of the cross-lagged path analysis.

	***Estimates/covariances***	***S.E*.**	**Standardized estimates (*r/β*)**	***p***
**Concurrent associations at the pretest**				
Positive youth development ^a^ ↔ life satisfaction	0.35	0.05	0.61	<0.001
Positive youth development ^a^ ↔ meaning in life	0.38	0.05	0.69	<0.001
Life satisfaction ↔ meaning in life	0.52	0.07	0.58	<0.001
**Temporal stability from the pretest to posttest**				
Positive youth development ^a^	0.52	0.08	0.52	<0.001
Life satisfaction	0.48	0.08	0.45	<0.001
Meaning in life	0.50	0.08	0.53	<0.001
**Cross-lagged effects from the pretest to posttest**				
Positive youth development ^a^ → life satisfaction	0.11	0.14	0.06	0.46
Positive youth development ^a^ → meaning in life	0.11	0.12	0.07	0.38
Life satisfaction → positive youth development ^a^	0.002	0.04	0.002	0.97
Life satisfaction → meaning in life	0.04	0.07	0.05	0.51
Meaning in life → positive youth development ^a^	0.11	0.05	0.17	0.03
Meaning in life → life satisfaction	0.21	0.09	0.19	0.02
**Correlated changes at the posttest**				
Positive youth development ^a^ ↔ life satisfaction	0.23	0.03	0.60	<0.001
Positive youth development ^a^ ↔ meaning in life	0.18	0.03	0.57	<0.001
Life satisfaction ↔ meaning in life	0.32	0.05	0.56	<0.001

a *Total score of positive youth development was sued*.

The three correlated changes were statistically significant (PYD competence and life satisfaction: *r* = 0.60, *p* < 0.001; MIL and PYD competence: *r* = 0.57, *p* < 0.001; MIL and life satisfaction: *r* = 0.56, *p* < 0.001), suggesting parallel improvements in students' wellbeing and MIL after the SL participation. Thus, Hypothesis 4 was supported.

## Discussion

Due to advances in information technology, there is growing adoption of online teaching and learning modes (e.g., blended learning and e-learning) in higher education settings in recent years. However, e-learning components have been less popular in service-learning (SL), possibly because SL is built upon an experiential learning pedagogy incorporating considerable interpersonal interactions. Nevertheless, mandatory social distancing measures against the spreading of COVID-19 have “forced” the transition of face-to-face teaching and learning to a non-face-to-face mode in SL. However, limited effort has been devoted to testing the effectiveness of online SL. It remains open as to whether the non-face-to-face SL experience involving online services during the COVID-19 pandemic is beneficial to student development. This study serves as a timely response to address this question and to explore the pathways underlying potential changes in wellbeing and MIL after non-face-to-face SL participation.

In the present study, students displayed significant positive changes in wellbeing, including life satisfaction and the overall PYD quality and its components such as cognitive and behavioral competence and positive identity. In line with most previous studies (50, 77), our results provide additional evidence for the hypothesis that SL experience would have a positive impact on students' wellbeing. Meanwhile, students also showed significant improvement in MIL, which is in line with the limited available literature on the role of SL participation in students' construction of MIL (35, 44, 45). The present findings may be explained by the argument that SL participation allows students to achieve self-development, social commitment, and deep reflection on life experience, which are important sources of MIL (41, 42).

The theory of SL highlights students' application of their professional knowledge and skills in serving the community, which would eventually benefit both the community and themselves (38). In traditional face-to-face SL, students provide face-to-face service in the community where they have deeper and closer interactions and relationships with their service recipients. However, during the COVID-19 pandemic, students could only conduct online services which might limit the effectiveness of their interaction with service recipients and their application of knowledge. Nevertheless, our findings suggest that SL participation in an online mode during the pandemic is also beneficial for university students' MIL and wellbeing. In other words, transforming SL from face-to-face mode to non-face-to-face mode may not necessarily hinder student learning achievement and development. This is consistent with previous studies showing adapted online subjects, including SL courses during the pandemic, remained as effective as pre-pandemic (52, 55, 82). In the current case, students interacted with teachers, service partners, and service targets through multiple platforms (e.g., email, phone calls, and social networking media) and they also committed to service design and delivery as previous students engaging in face-to-face SL. Such engagement and commitment may largely ensure the achievement of learning outcomes including both knowledge acquisition and skill application. In short, online SL is also beneficial for students' wellbeing and MIL.

Moreover, the improvement in MIL, life satisfaction, and PYD competence was significantly correlated with each other, suggesting that MIL and wellbeing improved hand in hand during the SL process. In the current non-face-to-face SL, students not only learned academic knowledge and service skills but also applied their knowledge and skills in serving children and adolescents in need through virtual learning platforms, during which they also had to deal with challenges and unexpected problems, such as technical issues, communication with different parties, and conflicts between group members. All these experiences may entail self-improvement, social commitment, a sense of helpfulness, feelings of self-worth and satisfaction, and deep reflection on life experience, which serve as essential sources of wellbeing and MIL (42, 69).

Despite a recent study showing significant reciprocal effects between MIL and wellbeing among Chinese university students (13), we only observed one-way significant cross-lagged effects of MIL on the two wellbeing indicators. Based on our findings, it seemed more likely that the enhancement in MIL during SL further contributed to the improvement in students' wellbeing rather than the opposite way. One possible explanation is that during the pandemic where there are uncertainties, suffering, death, and separation, life meaning is the foundation of wellbeing. At the same time, feeling satisfied with life and having positive mental health such as psychosocial competence may not be able to provide adequate meaning during the pandemic. As the present findings are not entirely consistent with the previous findings, it would be helpful to examine the issue and compare the findings during the pandemic and non-COVID-19 conditions further.

Two mechanisms may help explain the role of MIL in wellbeing. Primarily, according to the meaning maintenance model, the cognitive and emotional elements (e.g., clear and meaningful goals) integrated into an individual's sense of meaning and purpose directly promotes a feeling of wellbeing (70). On the other hand, MIL may promote other factors that contribute to one's wellbeing. For example, challenges and difficulties are inevitable during life transitions from adolescence to adulthood, and in transcending oneself in terms of focusing more on spiritual pursuit (e.g., cultivating oneself and helping others) rather than material urges. A clear sense of life meaning can serve as a strong intrinsic motivation that drives students to positively cope with these life challenges, hence enhancing university students' wellbeing (56, 71). Given limited evidence is available, more longitudinal studies are warranted to verify our speculations and explore the causality of the relationship between MIL and welbeing.

The present findings have important implications. Most scholars agree that SL (traditional face-to-face SL) is an important learning pedagogy that benefits not only needy people as service targets but also university students as service providers [i.e., the reciprocity of SL (38)]. This study provides additional evidence suggesting that non-face-to-face teaching and learning elements can be meaningfully incorporated into SL or other higher education curricula to effectively nurture students' life skills, life meaning, and wellbeing, even though these courses may involve intensive interpersonal interactions (52, 72, 78). The present findings inspire educators and youth workers to go beyond the traditional SL pedagogy and expand the scope of student engagement in community services.

First, non-face-to-face SL could be implemented even after the pandemic because of its convenience and cost-effectiveness. In particular, online SL makes it feasible to serve people living in remote places or overseas without traveling. For example, it would be exciting to develop SL projects for the left-behind children in developing countries where parents move to the city to earn a living. Likewise, people with reduced mobility can benefit from services at home, as long as these service targets have internet access. Moreover, such a pedagogy could be extended from the university sector to secondary school or even primary school settings. Indeed, SL has been empirically supported as a powerful teaching pedagogy for fostering students' intellectual, personal, social, and civic development from elementary school children to college students in the United States (40, 73). However, in Chinese communities such as Hong Kong, SL is a focus only in higher education. In the future, research efforts should be devoted to further investigating the effectiveness of online SL after the pandemic as well as beyond the higher education sector.

Despite the encouraging findings and potential implications, it is also important to note different challenges encountered in implementing non-face-to-face SL during the pandemic. For example, additional efforts are required to adjust curricula and train both teachers and students. In addition, service partners should be more involved to provide extra support as university students are not able to observe and communicate with service targets on-site. Moreover, online services might be less accessible for those needy people from low-income families who may be unable to afford digital devices and for those who lack digital literacy. In this case, support at the policy and institutional levels is essential for the future successful implementation of SL (both face-to-face and online SL) in all sectors related to education. For example, government sectors can set policies and guidelines to encourage schools to strategically incorporate SL in their school curricula and provide necessary resources and support. Meanwhile, government sectors, universities, and youth service sectors can jointly provide training programs for teachers to help them better understand SL and youth development theories as well as master SL teaching skills. Furthermore, extra support or resources should be deployed to both potential service providers and service recipients to increase the availability of online services.

The present study has several limitations. First, as the present study only focused on university students (i.e., service providers), educators and researchers need to further investigate whether other stakeholders (e.g., service targets) can also benefit from non-face-to-face SL and in what ways, such as testing the reciprocity view of SL and possible mechanisms involved (38). Second, without a control group, the present findings derived from the one-group pretest-posttest design might be attributable to alternative explanations (e.g., maturation) rather than the effect of the course. Future studies will certainly benefit from utilizing a quasi-experiment study that is more feasible than a randomized controlled trial in educational settings (74, 75).

Third, as we only included positive measures of wellbeing, it will offer illuminating insights if negative indicators, such as mental health problems (e.g., depression), are also included. Previous studies have indicated that a sense of meaning is a robust protector of youth mental health symptoms (14). Future research can examine whether SL experience in both face-to-face and non-face-to-face approaches can also help mitigate psychological distress among university students. Fourth, the effect size of the positive changes in MIL was not large and it was relatively lower than that of the increase in wellbeing. Some elements incorporated in SL, such as making contributions to community and society, may be especially essential for helping students find meaning (41). However, students might perceive less contribution in providing online services during the pandemic, which may hinder the increase in MIL. Nevertheless, more research is needed to identify what factors in SL experience are essential for promoting MIL. Finally, as the present data were obtained from self-report measures, findings might be affected by reporting bias. For example, students with better grades may tend to report higher self-evaluation on the measures. Although this is not necessarily the case as students did not know their course grades when they completed the questionnaires, future research can benefit from collecting additional data and investigating whether students' grades would affect the findings.

## Conclusion

Despite the limitations, the present study adds significant value by showing the positive impacts of online SL participation on university students' MIL and wellbeing during the COVID-19 pandemic. The findings reiterate that SL participation is beneficial for students despite the change of implementation mode from face-to-face to online. Together with previous positive findings obtained from traditional face-to-face SL programs, our findings suggest that SL is a promising method to promote participants' healthy development in normal times and in times of crisis such as during the pandemic in higher education and beyond. Furthermore, the current study expands the research scope by identifying simultaneous improvement in students' MIL and wellbeing as well as a significant contribution of MIL to wellbeing. This finding helps explain the mechanisms underlying the benefits of SL participation and further highlights the essential role of having a sense of meaning in living a good life.

## Data Availability Statement

The raw data supporting the conclusions of this article will be made available by the authors, without undue reservation.

## Ethics Statement

The studies involving human participants were reviewed and approved by the Human Subjects Ethics Sub-committee (HSESC) (or its Delegate) of The Hong Kong Polytechnic University. The patients/participants provided their written informed consent to participate in this study.

## Author Contributions

XZ contributed to the design of the project, data interpretation, and drafting and revising of the work. WC drafted and revised the work. DS conceived the project, obtained the funding, and edited the manuscript. LL collected data. All authors contributed to the article and approved the submitted version.

## Funding

The preparation of this article is financially supported by Wofoo Foundation.

## Conflict of Interest

The authors declare that the research was conducted in the absence of any commercial or financial relationships that could be construed as a potential conflict of interest.

## Publisher's Note

All claims expressed in this article are solely those of the authors and do not necessarily represent those of their affiliated organizations, or those of the publisher, the editors and the reviewers. Any product that may be evaluated in this article, or claim that may be made by its manufacturer, is not guaranteed or endorsed by the publisher.
